# 

**DOI:** 10.1192/bjb.2021.29

**Published:** 2021-12

**Authors:** Nidhi Gupta

**Affiliations:** Year 6 Specialty Trainee (ST6) in the Department of Adult Psychiatry, Birmingham and Solihull Mental Health NHS Foundation Trust, Birmingham, UK. Email: nidhigupta@doctors.net.uk

**Figure:**
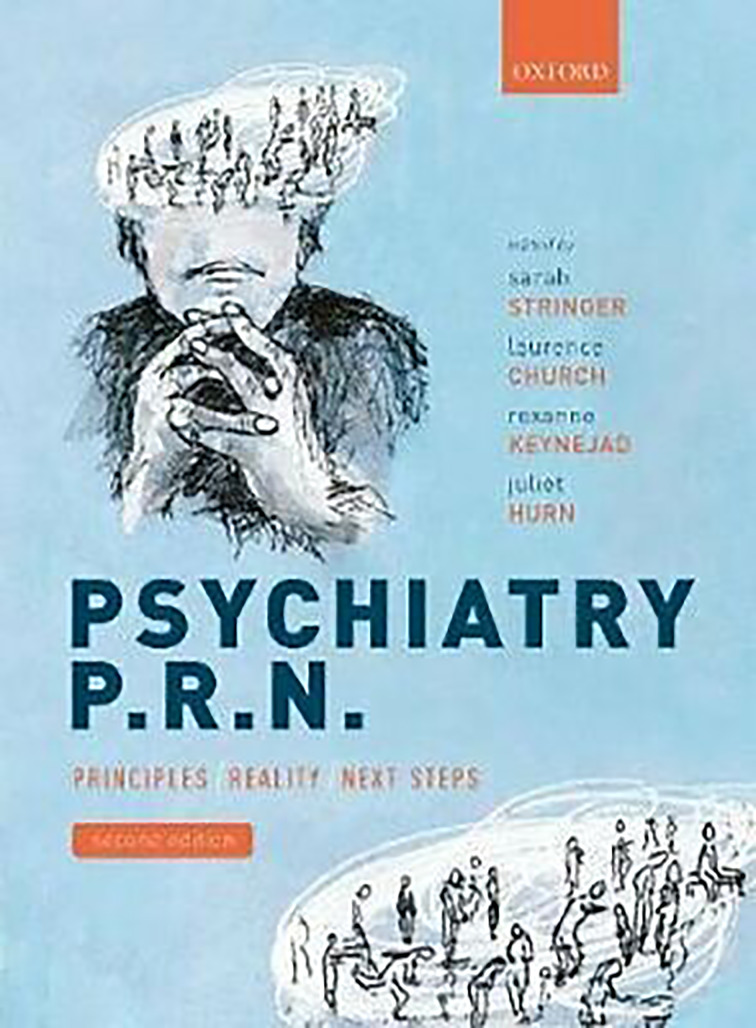

*Psychiatry P.R.N.* is edited by four psychiatrists who are involved in direct clinical care, and it shows in the way the book is configured to focus on practical, pragmatic learning. The use of multicoloured formatting, illustrations, text boxes and bullet points makes for easy reading and quick assimilation. Its small format also allows for easy portability in a handbag or backpack, to serve as a quick reference.

Though positioned as an undergraduate textbook for medical students, it will prove to be equally valuable to trainee psychiatrists, nurses, social workers and occupational therapists. Multiple choice question (MCQ) and objective and structured clinical examination (OSCE) skills are covered, case studies are aplenty and each chapter has salient points highlighted. At the end of each chapter, there is information available in the form of films, plays, novels, papers and useful resources. This second edition builds on the well-regarded first edition (2009), which was highly recommended in the BMA book awards (2010). The 2020 edition adds a chapter on forensic psychiatry. Chapters have been updated to incorporate new research and facts. Accessibility seems to have been a focus, with emphasis on diagrams, pictures and artwork.

The book is divided into two parts. Part 1 offers an overview of psychiatry, including psychiatric assessment, interview skills and psychiatry as a career choice. Part 2 focuses on ‘theory’, with an emphasis on imparting key information on major psychiatric conditions in a lucid, concise manner. Chapters are well laid out, with a uniform template that proffers developmental, neurobiological and sociological perspectives. Cross-cultural aspects of psychiatric illness are included in all the chapters.

I would have liked to see author credits for each chapter individually, but that is a minor quibble.

Strunk's *The Elements of Style*, first published a century ago, emphasised the rules of writing and language composition. *Psychiatry P.R.N.* reflects advancements in modern publishing, wherein the written word is intertwined with audio-visual and interactive media to enhance learning. I would suggest *Psychiatry P.R.N.* as recommended reading for all psychiatry training grades, as well as consultants who are interested in teaching and training.

